# Can we predict when to start renal replacement therapy in patients with chronic kidney disease using 6 months of clinical data?

**DOI:** 10.1371/journal.pone.0204586

**Published:** 2018-10-04

**Authors:** Min-Jeong Lee, Joo-Han Park, Yeo Rae Moon, Soo-Yeon Jo, Dukyong Yoon, Rae Woong Park, Jong Cheol Jeong, Inwhee Park, Gyu-Tae Shin, Heungsoo Kim

**Affiliations:** 1 Department of Nephrology, Ajou University School of Medicine, Suwon, Korea; 2 Department of Emergency Medicine, Ajou University School of Medicine, Suwon, Korea; 3 Department of Internal Medicine, Ajou University School of Medicine, Suwon, Korea; 4 Department of Biostatistics, Ajou University School of Medicine, Suwon, Korea; 5 Department of Medical Informatics, Ajou University School of Medicine, Suwon, Korea; Universidade Estadual Paulista Julio de Mesquita Filho, BRAZIL

## Abstract

**Purpose:**

We aimed to develop a model of chronic kidney disease (CKD) progression for predicting the probability and time to progression from various CKD stages to renal replacement therapy (RRT), using 6 months of clinical data variables routinely measured at healthcare centers.

**Methods:**

Data were derived from the electronic medical records of Ajou University Hospital, Suwon, South Korea from October 1997 to September 2012. We included patients who were diagnosed with CKD (estimated glomerular filtration rate [eGFR] < 60 mL·min^–1^·1.73 m^–2^ for ≥ 3 months) and followed up for at least 6 months. The study population was randomly divided into training and test sets.

**Results:**

We identified 4,509 patients who met reasonable diagnostic criteria. Patients were randomly divided into 2 groups, and after excluding patients with missing data, the training and test sets included 1,625 and 1,618 patients, respectively. The integral mean was the most powerful explanatory (R^2^ = 0.404) variable among the 8 modified values. Ten variables (age, sex, diabetes mellitus[DM], polycystic kidney disease[PKD], serum albumin, serum hemoglobin, serum phosphorus, serum potassium, eGFR (calculated by Chronic Kidney Disease Epidemiology Collaboration [CKD-EPI]), and urinary protein) were included in the final risk prediction model for CKD stage 3 (R^2^ = 0.330). Ten variables (age, sex, DM, GN, PKD, serum hemoglobin, serum blood urea nitrogen[BUN], serum calcium, eGFR(calculated by Modification of Diet in Renal Disease[MDRD]), and urinary protein) were included in the final risk prediction model for CKD stage 4 (R^2^ = 0.386). Four variables (serum hemoglobin, serum BUN, eGFR(calculated by MDRD) and urinary protein) were included in the final risk prediction model for CKD stage 5 (R^2^ = 0.321).

**Conclusion:**

We created a prediction model according to CKD stages by using integral means. Based on the results of the Brier score (BS) and Harrel’s C statistics, we consider that our model has significant explanatory power to predict the probability and interval time to the initiation of RRT.

## Introduction

The incidences of chronic kidney disease (CKD) and end-stage renal disease (ESRD) have been increasing rapidly [1]. The overall prevalence of CKD was found to be 8.2% in South Korea according to a study published in 2016 [2], and most patients with CKD have concerns about starting dialysis or undergoing transplantation. However, accurate prediction of the progression of disease and the timing of renal replacement therapy (RRT) remain problematic because of the lack of an accepted predictive tool for CKD progression that is effective and precise. In clinical practice, it is common for physicians to perform prognostic evaluation of a patient’s future disease progression based on a few recent measurements of glomerular filtration rate (GFR) or serum creatinine.

Therefore, physicians have difficulty in deciding which patients will ultimately progress to kidney failure and when they will require RRT. Identifying patients at risk of CKD progression may facilitate more optimal nephrology care. In the present study, we aimed to develop a model of CKD progression for predicting the probability and time to progression from CKD to RRT, using 6 months of clinical data variables routinely measured at healthcare centers. This developed model would provide more precise predictions than the commonly used Kidney Disease: Improving Global Outcomes (KDIGO) CKD stages, based eGFR and albuminuria.

## Material and methods

### Data source

The data were derived from the electronic medical record (EMR) database at Ajou University Hospital, Suwon, South Korea, from October 1997 to September 2012. This database contains information on patients and medical records, and includes data from all medical departments in the hospital. We extracted the data without personal identification to ensure patient confidentiality. The study was approved by the institutional review board of Ajou University Hospital.

### Study population

#### Study set

We included patients who were diagnosed with CKD and followed up for at least 6 months. The diagnostic criterion for CKD is estimated glomerular filtration rate (eGFR) < 60 mL·min^–1^·1.73 m^–2^ for ≥ 3 months [3]. The Modification of Diet in Renal Disease (MDRD) study equation or the Chronic Kidney Disease Epidemiology Collaboration (CKD-EPI) equation was used to calculate eGFR; We used both equations and included the patients if even one of the two equations(eGFR < 60 mL·min^–1^·1.73 m^–2^) was satisfactory. We excluded patients who were < 19 years old and those who had undergone RRT within 6 months of the study.

- MDRD equation
  $$186\times {serum\;creatinine}^{-1.154}\times {age}^{-0.203}\times 0.742\;(if\;female)$$
- CKD-EPI equation
  $$\begin{array}{l} 141\times \min{\left(\mathrm{Scr}/\kappa ,\;1\right)}^{\alpha }\times \max{\left(\mathrm{Scr}/\kappa ,\;1\right)}^{-1.209}\times 0.993^{\mathrm{Age}}\times 1.018\;\left(\mathrm{if}\;\mathrm{female}\right)\times 1.159\;\left(\mathrm{if}\;\mathrm{African}\right)\\ \;(\kappa =0.7\;\mathrm{if}\;\mathrm{female},\;\kappa =0.9\;\mathrm{if}\;\mathrm{male},\;\alpha =-0.329\;\mathrm{if}\;\mathrm{female},\\ \;\alpha =-0.411\;\mathrm{if}\;\mathrm{male},\;\min=\mathrm{minimum}\;\mathrm{Scr}/\kappa \;\mathrm{or}\;1,\\ \;\max=\mathrm{maximum}\;\mathrm{Scr}/\kappa \;\mathrm{or}\;1) \end{array}$$

#### Training set and test set

**Fig 1:**
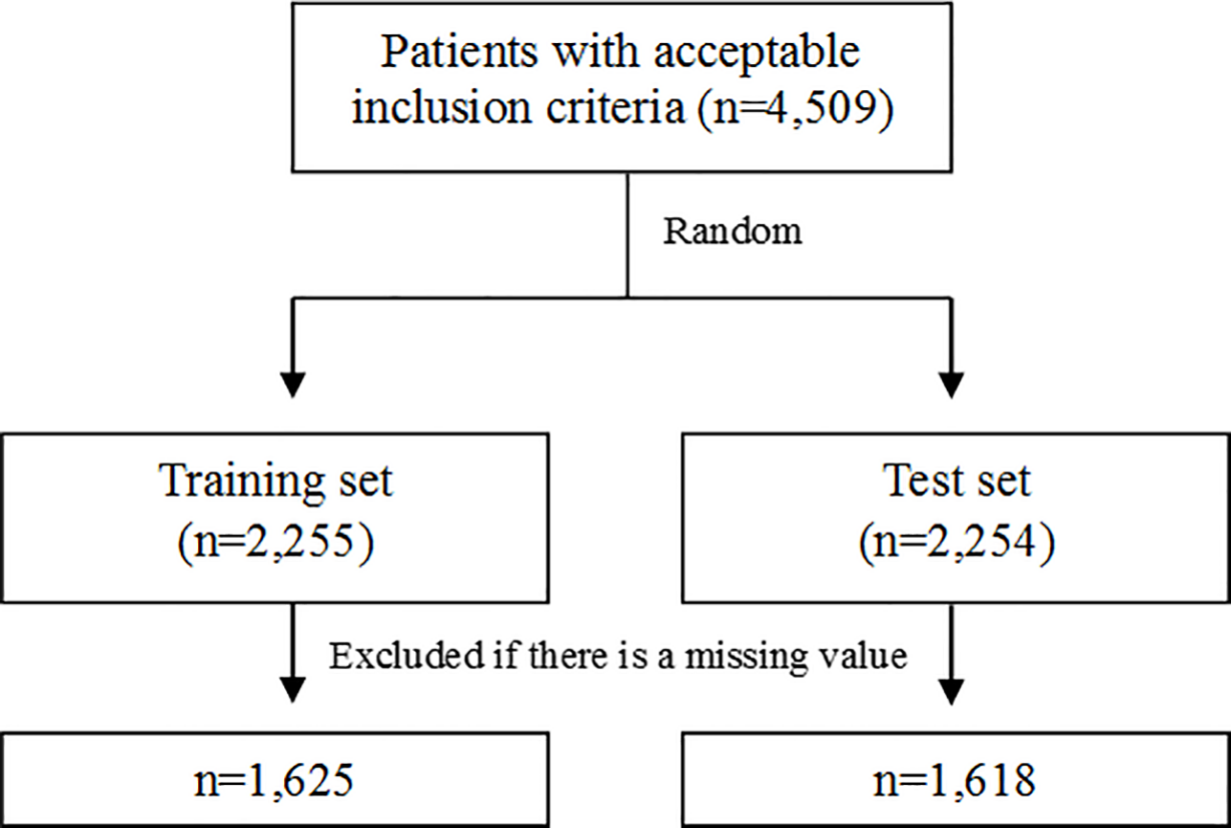
Flow diagram for patient’s selection.

We randomly divided the final study population into a training set and a test set for the verification process (Fig 1).

### Observation period and study period

The observation period was defined as interval from the initial day of observation to the day of initiation of RRT or the day of censoring. The initial day is the first day on which the eGFR decreased to < 60 mL·min^–1^·1.73 m^–2^. RRT included hemodialysis, peritoneal dialysis, and renal transplantation. The initial RRT point was defined as the first day of hemodialysis, day of catheter insertion for peritoneal dialysis, or the day of surgery for renal transplantation. If we could not identify the renal replacement event, we regarded the last follow-up date as the last observation day. The study period refers to the 180 days from the initial day of observation.

### Variables

The variables were as follows: demographic variables, including age and sex; comorbid conditions, including diabetes mellitus (DM), hypertension (HTN), glomerular nephritis (GN), systemic lupus erythematosus (SLE), and polycystic kidney disease (PKD); laboratory variables, including levels of blood urea nitrogen (BUN), hemoglobin, serum creatinine, serum calcium, serum phosphate, serum albumin, serum bicarbonate, urinary creatinine, urinary protein, and urinary blood, eGFR by the MDRD, and eGFR by the CKD-EPI. We excluded urine albumin, urine hemoglobin, and urine creatinine levels as variables because they were not measured in more than 50% of the patients.

Data regarding laboratory examination and comorbidity variables were collected throughout the study period. For missing values, we included data for 30 days before the initial day of observation and 30 days after completion of the study period. Urinary protein by dipstick was reported semi-quantitively as trace, 1+, 2+, 3+, or 4+ corresponding to albumin levels of 10, 30, 100, 300, or 1000mg/dl albumin respectively. Urinary protein level was coded as 5 dummy variables on the basis of negative values (trace, 1+, 2+, 3+, 4+). Criteria for the 5 comorbidities are described as follows.

1. DM: ICD-10 (E10–E14) code, serum HbA1c > 6.5%, or use of hypoglycemic medication
2. HTN: ICD-10 (I10–I15) code or use of antihypertensive medication
3. GN: ICD-10 (N01–N08) code
4. SLE: ICD-10 (M32) code
5. PCKD: ICD-10 (Q61) code

### Statistical analysis

#### Development of representative value

We developed 8 “modified values” that were potentially associated with CKD for 6 months and chose the “representative value” that demonstrated the greatest efficiency in a multivariate Cox proportional hazards regression model.

The modified values were: value at baseline, value at the end of the study period, minimum value, maximum value, ratio of the minimum to maximum values, slope of the minimum to maximum vales, integral means, and slope of initial to integral means(details as follows).

1. The value at baseline: the value obtained closest to the initial day of observation (± 30 days)
2. The value at the end of the study period: the value obtained closest to the end of the study period (± 30 days)
3. The minimum value: the minimum value during the study period
4. The maximum value: the maximum value during the study period
5. The ratio of the minimum to maximum values: the maximum value/minimum value
6. The slope of the minimum to maximum values
  $$\frac{Maximum\;value-Minimum\;value}{Day\;(maximum\;value)-Day\;(minimum\;value)}$$
7. The integral means
  $$\sum_{i=1}^{n-1}\frac{\left(b_{i}+b_{i+1})(a_{i+1}-a_{i}\right)}{2(a_{n}-a_{1})}$$
  (n = number of values, i = order, a = day of value recording, b = value on that day)
8. The slope of initial to integral means
  $$\frac{The\;integral\;means-The\;value\;at\;baseline}{Day\;(maximum\;value)-Day\;(minimum\;value)+90\;days}$$
  Values were excluded if 50% of the cases had missing data. Urinary protein(categorical variable) was only available at baseline.

#### Model development

Multivariate Cox proportional hazards regression was used for model development. We created a prediction model according to CKD stages [4]. The probability of the patient not undergoing RRT at time *t* (years) is as follows [5].

$$S(t)={S_{0}(t)}^{exp(\sum \beta_{i}\times X_{i}–\sum \beta_{i}\times {\tilde{u}}_{i})}$$

*t*: Followup time

*β*_*i*_: Regression coefficient

*X*_*i*_: Level of risk factor *i* of a patient

${\tilde{u}}_{i}:$ Corresponding average value of population

*S*_0_(*t*): Underlying probability of surviving

$“\sum \beta_{i}xX_{i}-\sum \beta_{i}x{\tilde{u}}_{i}”$ is defined as the risk index (RI): an increased value indicates a greater probability of RRT. We selected variables using clinical guidance and backward elimination (Wald) methods. The variables that did not contribute to the explanatory power of the RRT predictive model were removed until the remaining variables were significantly related to RRT (*p* < 0.05).

#### Evaluation of model performance

To evaluate the expected prediction error of the training model, we calculated the Brier score (BS) [6] and Harrel’s C statistics [7]. The BS is the square of deviation of the real value and the expected value. The higher the BS, the higher the expected error. If the BS is > 33%, the expected data show random levels, and if the BS is close to 0%, the expected data show perfect prediction.

Harrell’s C statistic is a common and well-validated measure to assess the discrimination. The higher the C-statistic, the better the model can discriminate between subjects who experience the outcome of interest and subjects who do not. C-statistics provide overall measures of predictive accuracy.

#### Software

We collected EMR data from Microsoft SQL Server 2012, and used PASW statistics (18.0.0) (SPSS Inc., Chicago, IL, USA) for selecting representative values. The multivariate Cox proportional hazards regression model, BS, and Harrel’s C statistics were analysed using R package (3.4.3).

## Results

### Patient selection

We identified 4,509 patients who met reasonable diagnostic criteria. Patients were randomly divided into 2 groups, and after the exclusion of patients with missing values, the training and test sets included 1,625 and 1,618 patients, respectively (Table 1).

**Table 1:** Baseline characteristics of the patients.

Characteristics		No. (%) of patients			*p* value
		Training set (*n* = 1,625)	Test set (*n* = 1,618)		
Demographics					
	Age (years)	60 (19–90)	59 (19–97)		NS
	Sex (male)	728 (44.8)	739 (45.7)		NS
Comorbidities					
	DM	640 (39.4)	626 (38.7)		NS
	HTN	610 (37.5)	562 (34.7)		.096
	GN	299 (18.4)	325 (20.1)		NS
	SLE	8 (0.5)	12 (0.7)		NS
	PKD	28 (1.7)	33 (2.0)		NS
Laboratory values					
	Serum albumin*(g/dL)	4.00 (1.44–5.33)	4.00 (1.49–5.10)		NS
	Serum creatinine*(mg/dL)	1.59 (0.63–13.50)	1.55 (0.76–14.25)		NS
	Serum hemoglobin*(g/dL)	11.75 (5.30–20.28)	11.78 (5.44–17.60)		NS
	Serum bicarbonate*(mEq/L)	24.00 (11.50–33.57)	23.97 (11.56–32.00)		NS
	Serum BUN*(mg/dL)	23.40 (6.60–145.85)	23.19 (6.25–142.67)		NS
	Serum calcium*(mg/dL)	8.93 (5.95–12.03)	8.90 (4.90–12.50)		NS
	Serum phosphorus*(mg/dL)	3.63 (1.00–8.99)	3.64 (1.33–9.40)		NS
	Serum potassium*(mEq/L)	4.50 (2.41–6.73)	4.50 (2.81–7.60)		NS
	eGFR (MDRD)*	39.89 (15.32)	41.07 (15.19)		.027‡
	eGFR (CKD-EPI)*	39.41 (15.65)	40.60 (15.55)		.031‡
	Urine protein^†^				NS
	Trace	151 (9.3)	162 (10.0)		
	1 positive	243 (15.0)	236 (14.6)		
	2 positive	438 (27.0)	402 (24.8)		
	3 positive	336 (20.7)	317 (19.6)		
	4 positive	57 (3.5)	58 (3.6)		
Outcome					
	Observation time	1096 (182–5089)	1306 (1003)		NS
	Renal replacement therapy events	530 (32.6)	473 (29.2)		.037‡

*Note*. χ^*2*^-test or Mann-Whitney U-test was used. Data are presented as number (%) or median (range)

*Integral means

^†^initial value.

‡*p*<0.05, *p* value above 0.10 replaced with “NS”(not significant).

### Set description

Patients in the training and test sets were similar with regard to demographics, comorbidities, laboratory values, and outcomes, with the exception of eGFR (MDRD), eGFR (CKD-EPI), and RRT events. The eGFR (MDRD) and eGFR (CKD-EPI) were lower (39.9 mL·min^–1^·1.73 m^–2^ vs. 41.1 mL·min^–1^·1.73 m^–2^ and 39.4 mL·min^–1^·1.73 m^–2^ vs. 40.6 mL·min^–1^·1.73 m^–2^, *p* < 0.05) and RRT events were higher (530/1,625 vs. 473/1,618, *p* < 0.05) in the training set than in the test set.

### Prediction model outcome

#### Representative values

We developed a multivariate Cox proportional hazards regression model with 8 modified values. We included 2,225 patients in the training set, and considered all collected variables. Eight modified values were all significantly effective, but the integral mean exhibited the most powerful explanatory value (R^2^ = 0.404), except for the end value (R^2^ = 0.546) (Table 2). We excluded the end value model because it included only 685 patients (< 50% of all patients). Thus, we used the integral mean as a representative value for each variable.

**Table 2:** Outcomes of model development using different modified values of variables.

Modified values	R^2^	Patients (n)
Baseline value	0.342	1,508
End value	0.546	685
Maximum	0.395	1,612
Minimum	0.373	1,612
Ratio of minimum to maximum	0.260	1,612
Integral mean	0.404	1,612
Slope of the minimum to maximum	0.244	1,168
Slope of initial to integral mean	0.298	1,508

#### Selection of prediction variables & outcome of the model

Variable selection process underwent through the multivariate Cox proportional hazards regression model using the backward elimination method. We made 3 models for three separate analysis for CKD stage 3–5. The final model for CKD stage 3 that included 10 selected variables (age, sex DM, PKD, levels of serum albumin, serum hemoglobin, serum phosphorous, and serum potassium, eGFR[CKD-EPI], and urinary protein) had risk predictive power of approximately 33% in the Cox proportional hazards regression model (Table 3). The risk is greater in patients who are female or elderly and in those who have DM. The greater the levels of serum albumin and eGFR (CKD-EPI), the lower the risk; the greater the levels of serum phosphorus and urine protein, the higher the risk. The model for CKD stage 4 that included 10 selected variables (age, sex, DM, GN, PKD, levels of serum haemoglobin, serum BUN, and serum calcium, eGFR[MDRD], and urinary protein) had risk predictive power of approximately 39% in the Cox proportional hazards regression model (Table 4). The risk is greater in patients who are female and in those who have PKD.

Table 5 shows the model for CKD stage 5 which had risk predictive power of approximately 32%. The model for CKD stage 5 included 4 selected variables, which was serum haemoglobin, serum BUN, eGFR[MDRD], and urinary protein.

**Table 3:** Regression coefficients and hazard ratios for variables in the risk prediction model at CKD stage 3 patients.

	Training set					
	Regression coefficient	SE	HR	95% CI for HR		*p* value
				Lower	Higher	
**Age (years)**	–0.024***	0.06	0.98	0.97	0.99	<0.001
**Sex (male)**	–0.424**	0.14	0.65	0.50	0.86	0.002
**DM**	0.527***	0.14	1.69	1.28	2.24	<0.001
**PKD**	1.068	0.62	2.91	0.89	9.56	0.079
**Serum albumin**	–0.976***	0.14	0.38	0.29	0.50	<0.001
**Serum hemoglobin**	–0.078	0.05	0.93	0.85	1.01	0.093
**Serum phosphorus**	0.422***	0.10	1.53	1.24	1.87	<0.001
**Serum potassium**	0.217	0.15	1.24	0.93	1.65	NS
**eGFR(CKD-EPI)**	–0.050***	0.01	0.95	0.94	0.96	<0.001
**Urine protein, 1+**	1.085***	0.27	2.96	1.73	5.07	<0.001
**Urine protein, 2+**	1.253***	0.24	3.50	2.18	5.62	<0.001
**Urine protein, 3+**	1.289***	0.26	3.63	2.19	6.02	<0.001
**Urine protein, 4+**	1.520***	0.36	4.57	2.27	9.22	<0.001

*Note*. R^2^ = 0.330, *p* <0.001.

HR = hazard ratio, CI = confidence interval.

*p<0.05

**p<0.01

***p<0.001, *p* value above 0.10 replaced with “NS”(not significant).

**Table 4:** Regression coefficients and hazard ratios for variables in the risk prediction model at CKD stage 4 patients.

	Training set					
	Regression coefficient	SE	HR	95% CI for HR		*p* value
				Lower	Higher	
**Age (years)**	–0.010	0.01	0.99	0.98	1.00	NS
**Sex (male)**	–0.433*	0.50	0.65	0.44	0.95	0.027
**DM**	0.397	0.21	1.49	0.98	2.25	0.059
**GN**	-0.318	0.22	0.73	0.47	1.12	NS
**PKD**	1.786**	0.56	5.60	2.00	17.81	0.001
**Serum hemoglobin**	–0.109	0.07	0.90	0.78	1.03	NS
**Serum BUN**	–0.016	0.01	0.98	0.97	1.00	NS
**Serum calcium**	–0.318	0.19	0.73	0.50	1.06	NS
**eGFR(MDRD)**	–0.107***	0.02	0.90	0.87	0.93	<0.001
**Urine protein, 1+**	1.211**	0.42	3.36	1.46	7.70	0.004
**Urine protein, 2+**	1.279**	0.39	3.59	1.67	7.72	0.001
**Urine protein, 3+**	1.853***	0.41	6.38	2.88	14.16	<0.001
**Urine protein, 4+**	2.539***	0.56	12.67	4.23	37.89	<0.001

*Note*. R^2^ = 0.386, *p*<0.001.

HR = hazard ratio, CI = confidence interval.

*p<0.05

**p<0.01

***p<0.001, *p* value above 0.10 replaced with “NS”(not significant).

**Table 5:** Regression coefficients and hazard ratios for variables in the risk prediction model at CKD stage 5 patients.

	Training set					
	Regression coefficient	SE	HR	95% CI for HR		*p* value
				Lower	Higher	
**Serum hemoglobin**	–0.100	0.06	0.91	0.81	1.02	0.097
**Serum BUN**	0.015	0.01	1.02	1.00	1.03	0.013
**eGFR(MDRD)**	–0.070**	0.02	0.93	0.89	0.98	0.002
**Urine protein, 2+**	0.669*	0.29	1.95	1.11	3.43	0.019
**Urine protein, 3+**	0.916**	0.41	2.50	1.40	4.47	0.002
**Urine protein, 4+**	0.988	0.56	2.69	0.90	8.06	0.078

*Note*. R^2^ = 0.321, *p*<0.001.

HR = hazard ratio, CI = confidence interval.

*p<0.05

**p<0.01

***p<0.001.

#### Risk prediction model

The risk index (RI) of CKD stage 3 patients can be defined as follows.

$$\begin{array}{l} \mathrm{RI}\\ =-0.002\times \mathrm{age}\left(\mathrm{years}\right)-0.976\times \mathrm{albumin}\left(g/\mathrm{dL}\right)-0.078\times \mathrm{hemoglobin}\left(g/\mathrm{dL}\right)\\ +0.422\times \mathrm{phosphorus}\left(\mathrm{mg}/\mathrm{dL}\right)+0.217\times \mathrm{potassium}\left(\mathrm{mEq}/L\right)-0.050\\ \times \mathrm{eGFR}\left(\mathrm{CKD}\;\mathrm{EPI}\right)+5.968-0.424\;\left(\mathrm{if}\;\mathrm{female}\right)+0.527\left(\mathrm{if}\;\mathrm{DM}\;\mathrm{is}\;\mathrm{present}\right)\\ +1.068\left(\mathrm{if}\;\mathrm{PKD}\;\mathrm{is}\;\mathrm{present}\right)+1.085\left(\mathrm{if}\;\mathrm{urine}\;\mathrm{protein}=1+\right)\\ +1.253\left(\mathrm{if}\;\mathrm{urine}\;\mathrm{protein}=2+\right)+1.289\left(\mathrm{if}\;\mathrm{urine}\;\mathrm{protein}=3+\right)\\ +1.520\left(\mathrm{if}\;\mathrm{urine}\;\mathrm{protein}=4+\right) \end{array}$$

The RI of CKD stage 4 patients can be defined as follows.

$$\begin{array}{l} \mathrm{RI}\\ =-0.002\times \mathrm{age}\left(\mathrm{years}\right)-0.976\times \mathrm{albumin}\left(g/\mathrm{dL}\right)-0.078\times \mathrm{hemoglobin}\left(g/\mathrm{dL}\right)\\ +0.422\times \mathrm{phosphorus}\left(\mathrm{mg}/\mathrm{dL}\right)+0.217\times \mathrm{potassium}\left(\mathrm{mEq}/L\right)-0.050\\ \times \mathrm{eGFR}\left(\mathrm{CKD}\;\mathrm{EPI}\right)+5.968-0.424\;\left(\mathrm{if}\;\mathrm{female}\right)+0.527\left(\mathrm{if}\;\mathrm{DM}\;\mathrm{is}\;\mathrm{present}\right)\\ +1.068\left(\mathrm{if}\;\mathrm{PKD}\;\mathrm{is}\;\mathrm{present}\right)+1.085\left(\mathrm{if}\;\mathrm{urine}\;\mathrm{protein}=1+\right)\\ +1.253\left(\mathrm{if}\;\mathrm{urine}\;\mathrm{protein}=2+\right)+1.289\left(\mathrm{if}\;\mathrm{urine}\;\mathrm{protein}=3+\right)\\ +1.520\left(\mathrm{if}\;\mathrm{urine}\;\mathrm{protein}=4+\right) \end{array}$$

The RI of CKD stage 5 patients can be defined as follows.

$$\begin{array}{l} RI\\ =-0.098\times hemoglobin(g/dL)+0.015\times BUN(mg/dL)-0.070\times eGFR(MDRD)\\ +1.039+0.669(if\;urine\;protein=2+)+0.916(if\;urine\;protein=3+)\\ +0.988(if\;urine\;protein=4+) \end{array}$$

By using RI, the formula for the probability of a patient not undergoing RRT at some point (*t*, years) is as follows.

$$S(t)={S_{0}(t)}^{exp(RI)}$$

*S*(*t*): probability of not undergoing renal replacement therapy

*S*_0_(*t*): underlying probability

(*S*_0_(1) = 0.973,*S*_0_(3) = 0876,*S*_0_(5) = 0.756,*S*_0_(7) = 0.756,*S*_0_(10) = 0.423)

### Test set

#### Brier score

To evaluate the expected prediction error of the training set model, we calculated the weighted BS that gave the weighted value to censored data. The period during which the BS is < 0.33 is approximately 5,000 days at the model of CKD stage 3 and 5. The period during which the BS < 0.33 is approximately 4,000 days at the model of CKD stage 4. Thus, the prediction model gives a marginal predictive result up to approximately 4,000–5,000days (S1–S3 Figs).

#### Harrel’s C statistics

To evaluate the accuracy of the prediction model, we calculated Harrel’s C statistics. The C-statstics of final model was 0.86 (0.83–0.88) at CKD stage 3, 0.80 (0.76–0.84) at CKD stage 4, 0.84 (0.78–0.90), respectively.

#### Example cases of prediction model application

**Fig 2:**
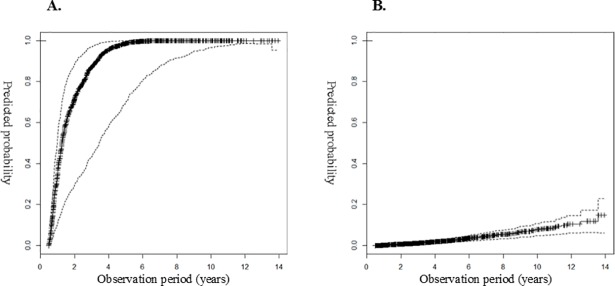
Predicted probability of starting renal replacement therapy. A) Patient with RRT after 5 years of follow-up (age = 56 years, sex = female, DM = no, PKD = yes, albumin = 4.06g/dl^a^, hemoglobin = 8.01g/dl^a^, calcium = 8.31mg/dl^a^, phosphorus = 3.16mg/dl^a^, potassium = 4.95mmol/L^a^, eGFR = 18.36, protein = 2+). B) Patient without RRT censored after 5 years of follow-up (age = 58 years, sex = male, DM = yes, PKD = no, albumin = 4.65g/dl^a^, hemoglobin = 12.11g/dl^a^, calcium = 9.70mg/dl^a^, phosphorus = 3.10mg/dl^a^, potassium = 4.02mmol/L^a^, eGFR = 54.81, protein = negative). (^a^integral mean value) Side lines are 95% confidence interval (CI).

We analysed 2 cases in which the observation period was approximately 5 years, using the risk prediction model in the test set. Fig 2A shows the graph for the probability of the event for a 56-year-old female patient who experienced progression to RRT after 5 years. The probability of the event was > 80% at 3 years and > 95% at 5 years. Fig 2B shows the probability of the event in a 58-year-old male patient who did not experience progression to RRT after 5 years. The probability of an event was < 20% at 10 years.

## Discussion

CKD is asymptomatic in the early stages, but symptoms appear in the later stages, accompanied by complications such as cardiovascular disease, anemia, infection, cognitive impairment, and impaired physical function [8–11]. The KDIGO clinical practice guideline suggested a prognostic classification system for CKD divided on the basis of 6 categories of GFR, 3 categories of albuminuria stage, and cause of disease. Based on these findings, KDIGO devised 3 broad risk categories based upon the likelihood of developing future kidney and cardiovascular complications [12]. However, eGFR assessment and ascertainment of albuminuria may not be sufficient for risk prediction in the clinic.

We considered many variables cited in previous articles that could affect renal function, including age, sex, laboratory findings, and comorbidities, to develop a risk prediction model. These included variables such as young age, male sex, African-American ethnicity, DM, HTN, obesity, urine protein, serum albumin, anemia, lipidemia, smoking, and cardiovascular disease [13]. In the Reduction of Endpoints in NIDDM with the Angiotensin II Antagonist Losartan (RENAAL) study, albuminuria, hypoalbuminemia, increased serum creatinine, and decreased hemoglobin were the risk factors associated with ESRD in patients with type 2 DM and nephropathy [14]. We collected data on the above variables, and identified data that were not measured in > 50% of the patients. Our study was performed retrospectively in order to identify missing variables that could significantly affect RRT.

We identified variables that were associated with RRT through the clinical guidance and backward elimination (Wald) methods. From a clinical point of view, models bsased on referral eGFR are more useful than an overall model. Predictions for patient with an eGFR of 60 would probably only be interesting to the patient, while predictions for a patient with an eGFR 15 are critical for dialysis preparation. So we underwent separate analysis according to CKD stages: 1) CKD stage 3: age, sex, DM, PKD, levels of serum albumin, serum hemoglobin, serum phosphate, and serum potassium, eGFR, and urinary protein. 2) CKD stage 4: age, sex, DM, GN, PKD, levels of serum hemoglobin, serum BUN, serum calcium, eGFR, and urinary protein. 3) CKD stage 5: level of serum hemoglobin, serum BUN, eGFR, and urinary protein. The results were similar to those of previous studies. First, one study reported that the risk of progression to ESRD was decreased among older patients with CKD stage 3 (hazard ratio [HR], 0.75; 95% confidence interval, 0.63–0.89 for each 10-year increase in age) [15]. Second, another study showed that male patients with CKD stage 4 and 5 had a shorter time to RRT than did female patients [16]. Third, it is thought that DM is rapidly becoming the most common cause of ESRD and is also associated with an increasing risk of ESRD [17]. In the African American Study of Kidney Disease and Hypertension (AASK) trial, the change in urinary protein level from baseline to 6 months predicted progression to RRT [18]. In the RENAAL study, baseline hemoglobin was an important independent variable for prediction of ESRD among diabetic patients [19]. Moreover, HTN has been found to be predictive of ESRD risk in several large population-based studies [17, 20]. However, the presence of HTN was not an independent predictor of kidney failure events in the present study. The RENAAL study showed similar findings, a result likely due to the fact that blood pressure was well controlled in the study patients [14].

To identify representative values that show renal function change over 6 months, we considered 8 modified values and developed a multivariate Cox proportional hazards regression model. The integral mean contains the time and the value in order to obtain sufficient power to explain the change in data over 6 months. The end value had the highest R^2^, but the number of patients was inadequate to evaluate the model. We will compare the integral mean and end value in a larger dataset in a further study.

Finally, we developed the renal prediction model with several variables using integral means from continuous variables. To evaluate prediction error, we calculated the BS and the Harrel’s C statistics. From the results of the BS and Harrel’s C statistics, we consider that our model has sufficient explanatory power to predict renal progression.

The strength of our analysis is that we divided patients into 2 groups: the training set and the test set. Thus, we calculated the BS and Harrel’s C statistics in order to confirm the accuracy of the model. Second, the prediction equation must include variables that are very routinely available in the nephrology clinic for convenience of use. Local healthcare facilities can collect laboratory data easily and integrate the risk prediction tool into decision-making for patients who require further evaluation or in preparation for RRT.

The limitations of our analysis are that the study was performed retrospectively, and therefore, the data obtained are insufficient including blood pressure measurements of the patients, which was important predictor in previous studies [17, 20]. We considered many variables from previous studies while developing the risk prediction tool, but insufficient data were available for evaluation from the EMR. Second, patients with missing data were excluded. Since missing data are usually selectively missing this causes a selection bias. Third, all of our study subjects were Asian, especially Korean, there is a limitation about applicability of the results in other occidental countries. Fourth, there is no standard procedure for determining the initiation of RRT; therefore, initiation of therapy may reflect personal opinions, and patients’ economic, social, and environmental factors may also affect the timing. However, the selection of the test set and the training set from the same hospital in the present study meant that the prediction error was reduced because the characteristics of patients in the training set and test set were similar. Fifth, our study is a lack of renal diagnosis. Because we collected data not from accurate chart review but from the EMR, we have not been able to present the primary cause of ESRD. Instead, we considered comorbidities with high prevalence as the primary cause of ESRD in Korea [21].

Many studies have identified a wide range of risk factors for the progression of CKD. Although many studies have identified similar risk factors, there has not been sufficient research performed on the risk prediction models for RRT. To develop accurate and easy-to-use models, further large prospective studies are required. Our predictive model for CKD may have sufficient power to predict RRT, as shown in 2 cases in the present study. However, there are also cases that did not fit the model. If data were collected from a greater number of patients with greater accuracy, a more precise model could be developed. The development of the representative value seems very complicated for a prediction tool in a clinical setting. To simplify this model could be achieved by cooperation among nephrologists and statisticians.

In summary, a model was developed and validated to predict the risk for ESRD. This model uses commonly available clinical variables and may -provide more precise predictions than the commonly used KDIGO CKD stages, based on eGFR and albuminuria.

## Supporting information

S1 Fig Prediction error according to the observation period (days) at CKD stage 3. (EMF)

S2 Fig Prediction error according to the observation period (days) at CKD stage 4. (EMF)

S3 Fig Prediction error according to the observation period (days) at CKD stage 5. (EMF)

S1 Table Modeling dataset. (CSV)

S2 Table Validation dataset. (CSV)
